# Supplementary material for: Structure of CFTR bound to (R)-BPO-27 unveils a pore-blockage mechanism
Source: Nat Commun. 2025 Aug 1;16:7059. doi: 10.1038/s41467-025-62199-7 (PMC12317126; doi:10.1038/s41467-025-62199-7)
Supplement: Supplementary file 1 — Supplementary Information [file 41467_2025_62199_MOESM1_ESM.pdf]

Structure of CFTR bound to (*R*)-BPO-27 unveils a pore-blockage mechanism

Paul G. Young[1,2], Karol Fiedorczuk[1], & Jue Chen[1,3,*]

[1]Laboratory of Membrane Biology and Biophysics, The Rockefeller University, New York, NY 10065

[2]Weill Cornell/Rockefeller/Sloan Kettering Tri-Institutional MD-PhD Program, New York, NY 10065

[3]Howard Hughes Medical Institute, The Rockefeller University, 1230 York Ave, New York, NY 10065

*To whom correspondence should be addressed: juechen@rockefeller.edu.

Keywords: CFTR, ion channel, inhibitor, pore blocker

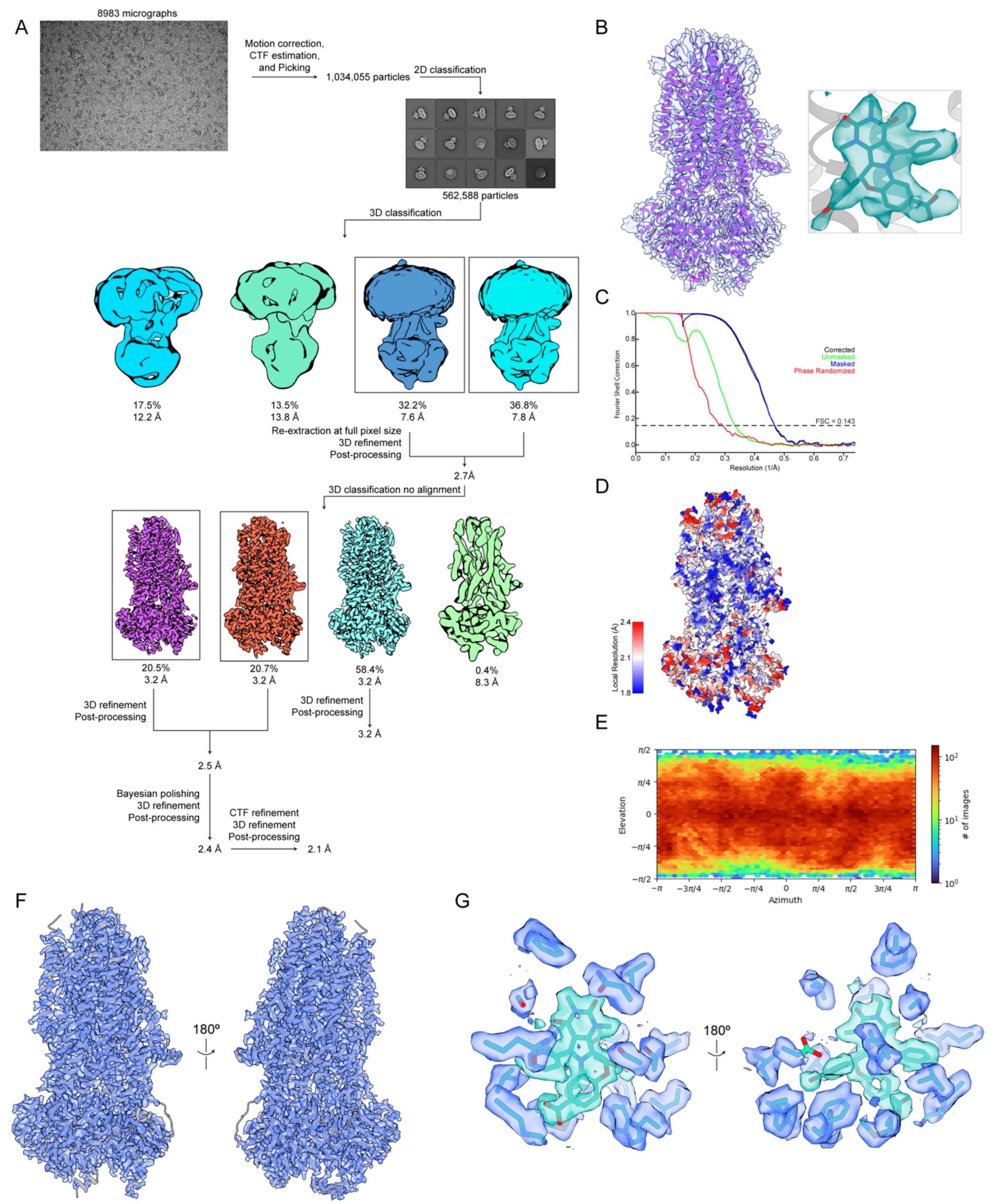

Figure S1. Cryo-EM analysis of the CFTR/(*R*)-BPO-27 complex and quality of the reconstruction.

(A) Image processing procedure.

(B) (*Left*) Superposition of final model (magenta) with the 58%-occupancy class (shown as blue surface) from the second round of 3D classification after 3D refinement and postprocessing. This class represents lower quality particles, rather than a unique conformation. (*Right*) Density corresponding to (*R*)-BPO-27 is present in this class. (*R*)-BPO-27 modeled into the density in this class (shown as teal surface).

(C) Fourier shell correlation curves of the final map.

(D) Local resolution estimation of the final map.

(E) Euler angle particle distribution.

(F) Two views of the atomic model fit into the final map at a contour level of 0.00789.

(G) Cryo-EM density of (*R*)-BPO-27 and residues within 4.5 Å fit with the atomic model. The map is shown at a contour level of 0.00597.

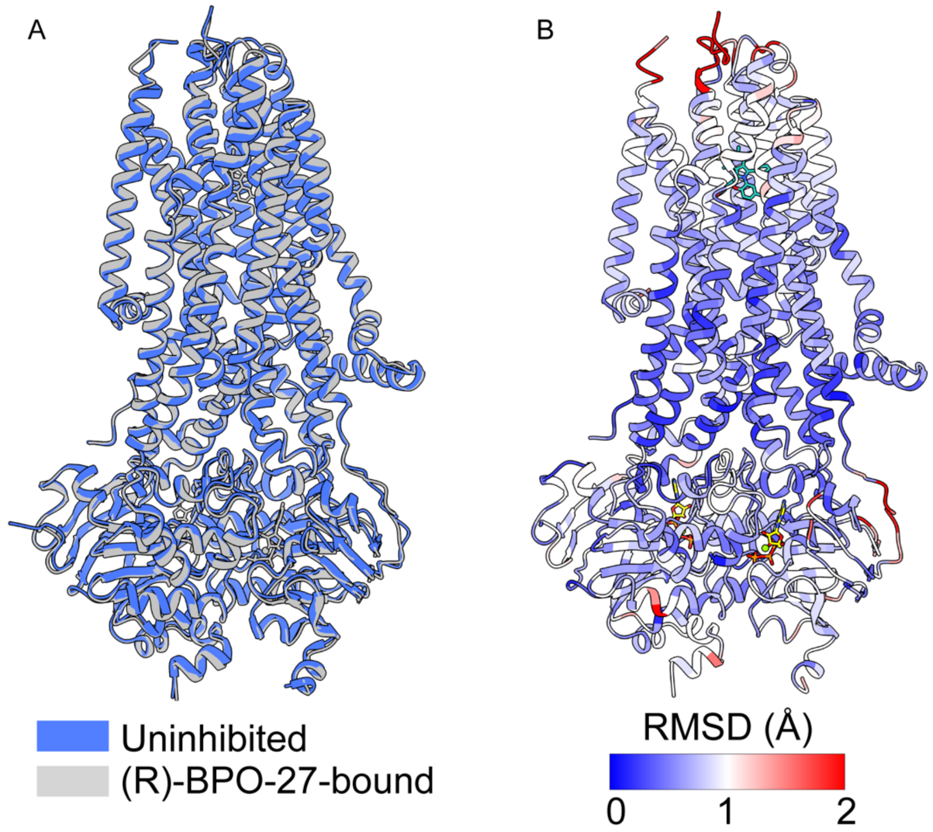

Figure S2. The conformation of the CFTR/(*R*)-BPO-27 complex is similar to uninhibited structures.

(A) Superposition of uninhibited, dimerized CFTR (blue) with the CFTR/(*R*)-BPO-27 complex (gray).

(B) Structure of the CFTR/(*R*)-BPO-27 complex colored by RMSD from the uninhibited structure.

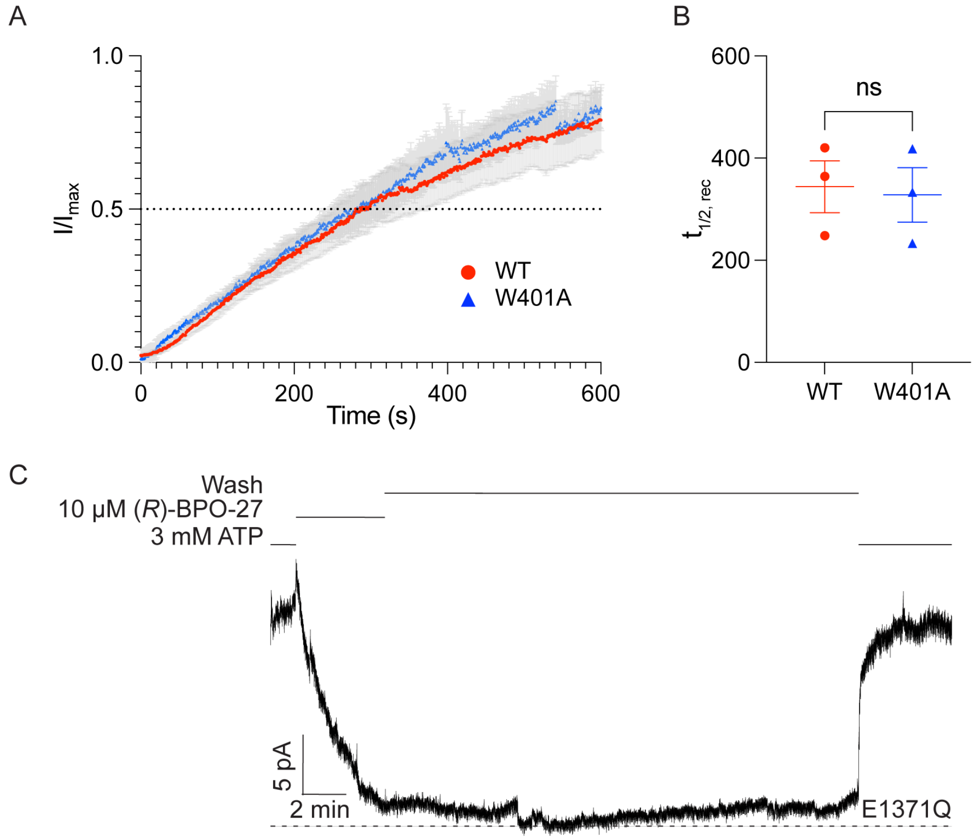

Figure S3. (*R*)-BPO-27 dissociation requires NBD dissociation.

(A) Normalized plots of current recovery after withdrawal of 10 μM (*R*)-BPO-27 and application of 3 mM ATP for WT CFTR and CFTR (W401A). The dotted line indicates 50% maximal current. Data represent means and SE for n=3 measurements.

(B) Quantification of recovery $t_{1/2}$ values for WT CFTR and CFTR (W401A). For each replicate, $t_{1/2}$ values were determined based on the time at which half-maximal current was achieved. Mean $t_{1/2}$ and SE were then calculated across replicates. Data represent means and SE for n=3 measurements. Significance was tested using a two-tailed Student's t-test ($P = 0.84$).

(C) Representative macroscopic current recording from an inside-out membrane patch excised from cells expressing CFTR (E1371Q). CFTR was fully phosphorylated by PKA in the presence of 3 mM ATP before the displayed recording.

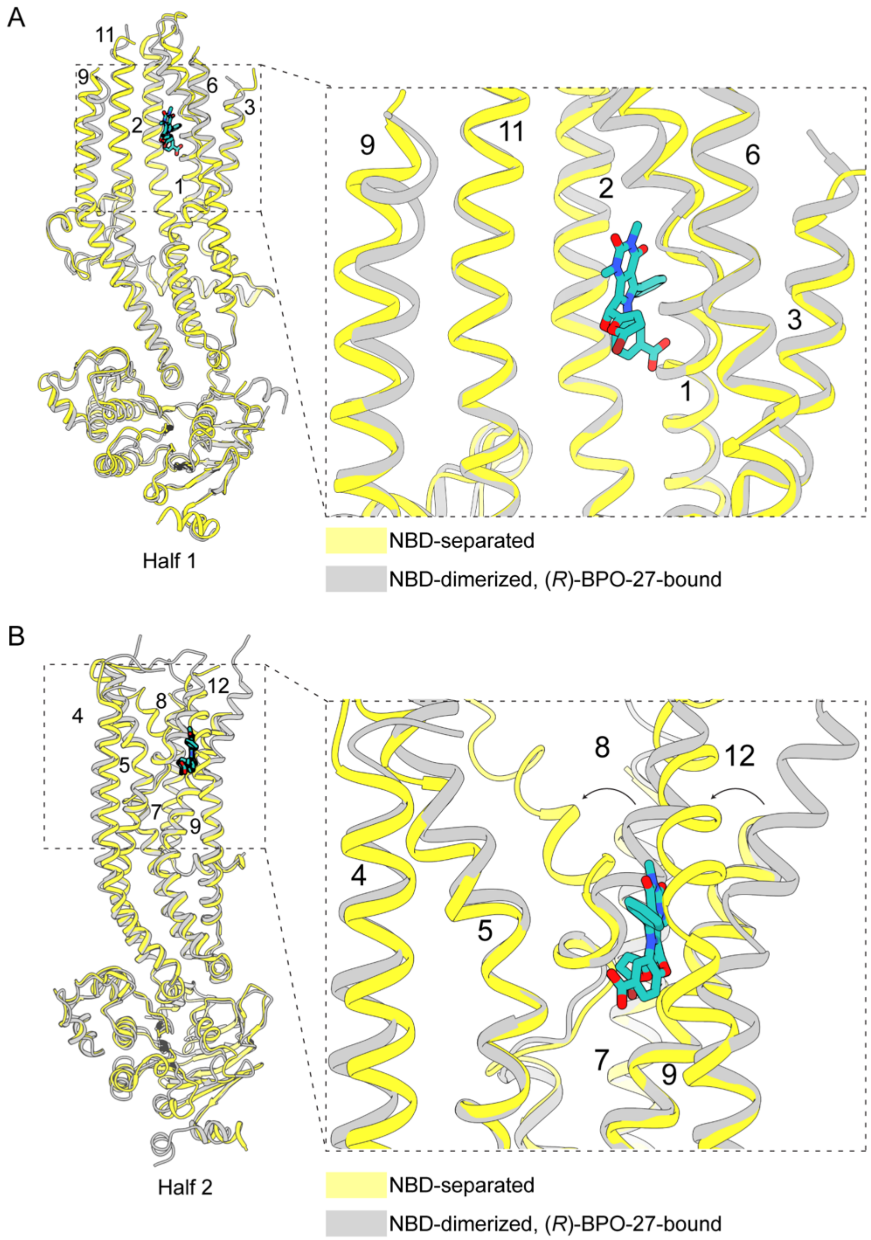

Figure S4. NBD separation perturbs the (*R*)-BPO-27 binding site.

(A) (*Left*) Superposition of the NBD1-containing halves of CFTR from the NBD-separated structure (PDB: 5UAK) and the (*R*)-BPO-27-bound structure. (*R*)-BPO-27 is modeled as teal sticks into its binding site. (*Right*) Close-up view of the (*R*)-BPO-27 binding site.

(B) (*Left*) Superposition of the NBD2-containing halves of CFTR from the NBD-separated structure (PDB: 5UAK) and the (*R*)-BPO-27-bound structure. (*R*)-BPO-27 is modeled as teal sticks into its binding site. (*Right*) Close-up view of the (*R*)-BPO-27 binding site. Arrows reflect the direction of movement of TMs 8 and 12 from their positions in the (*R*)-BPO-27-bound structure to their positions in the NBD-separated structure.

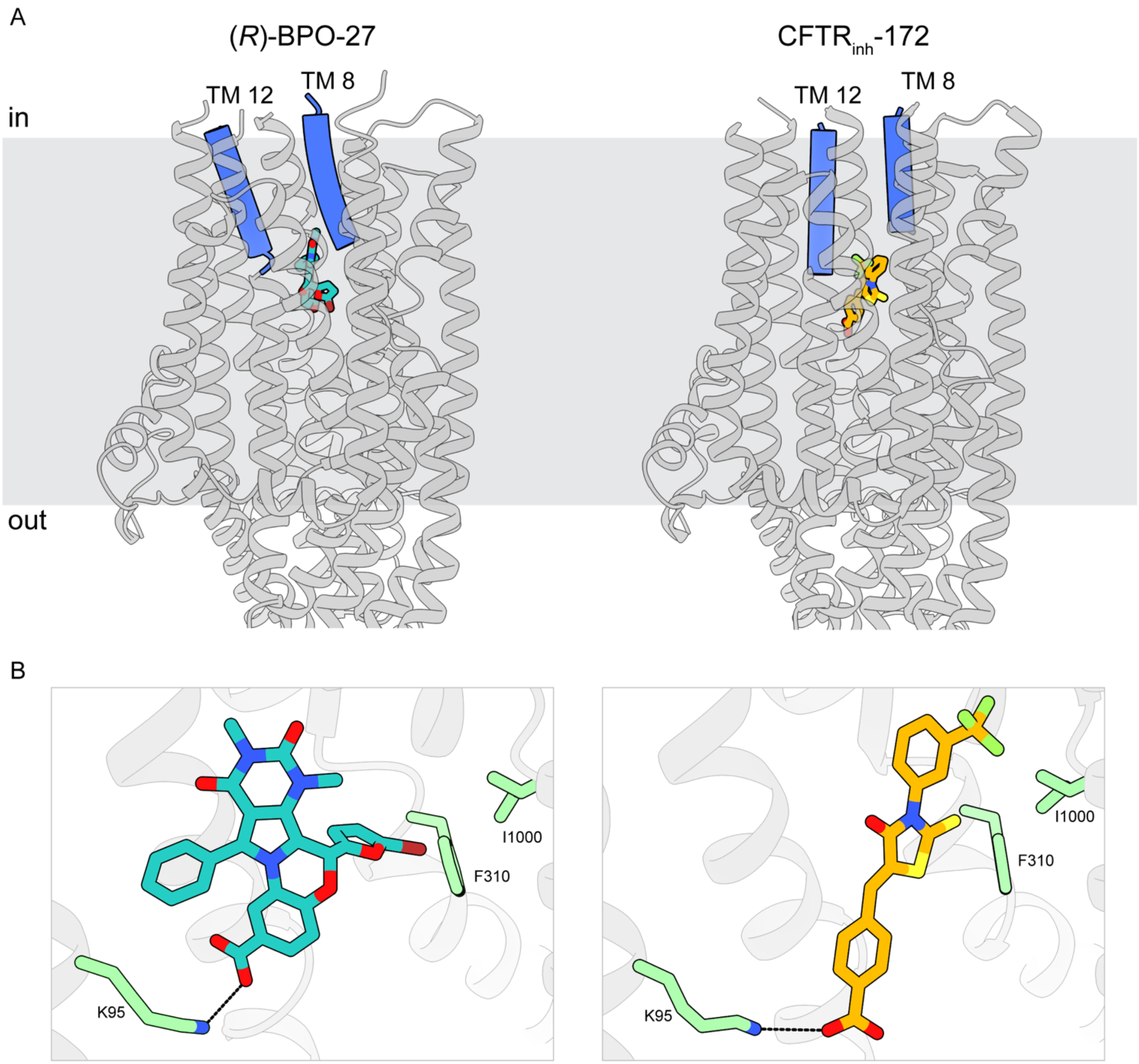

Figure S5. (*R*)-BPO-27 and CFTR$_{inh}$-172 bind similar sites within the pore but stabilize different conformational states.

(A) Transmembrane regions of CFTR/(*R*)-BPO-27 and CFTR/CFTR$_{inh}$-172 (PDB: 8UBR) complexes. Inhibitors are shown as sticks. Helices stabilized in a unique conformation in the CFTR$_{inh}$-172-bound structure are shown as blue cylinders in both models.

(B) Comparison of the binding sites of (*R*)-BPO-27 (*Left)* and CFTR$_{inh}$-172 (Right). Inhibitors are shown as sticks. Three residues that form part of the binding site are shown.

	CFTR/(*R*)-BPO-27 + ATP<br>(EMD-48717)<br>(PDB 9MXL)
**Data collection and processing**	
Magnification	105,000
Voltage (kV)	300
Electron exposure (e^-/Å^2)	65.6
Defocus range (μm)	0.5-2.5
Pixel size (Å)	0.676
Symmetry imposed	C1
Initial particle images (no.)	1034355
Final particle images (no.)	160342
Map resolution (Å)	2.1
FSC threshold	0.143
Map resolution range (Å)	1.5-2.9
**Refinement**	
Initial model used (PDB code)	6MSM
Model resolution (Å)	2.32
FSC threshold	0.5
Map sharpening *B* factor (Å^2)	-30.824
Model composition	
Non-hydrogen atoms	9582
Protein residues	1190
Ligands	8
B factors (Å^2)	
Protein	79.55
Ligand (inhibitor)	48.9
R.m.s. deviations	
Bond lengths (Å)	0.005
Bond angles (º)	0.670
Validation	
MolProbity score	1.16
Clashscore	3.67
Poor rotamers (%)	0.40
Ramachandran plot	
Favored (%)	98.30
Allowed (%)	1.70
Disallowed (%)	0

Table S1. Statistics for the CFTR/(*R*)-BPO-27 complex atomic model.
